# Interacting Social Processes on Interconnected Networks

**DOI:** 10.1371/journal.pone.0163593

**Published:** 2016-09-30

**Authors:** Lucila G. Alvarez-Zuzek, Cristian E. La Rocca, Federico Vazquez, Lidia A. Braunstein

**Affiliations:** 1 IFIMAR, Instituto de Investigaciones Físicas de Mar del Plata (CONICET-UNMdP), 7600 Mar del Plata, Argentina; 2 IFLYSIB, Instituto de Física de Líquidos y Sistemas Biológicos (CONICET-UNLP), 1900 La Plata, Argentina; Semmelweis University, HUNGARY

## Abstract

We propose and study a model for the interplay between two different dynamical processes –one for opinion formation and the other for decision making– on two interconnected networks *A* and *B*. The opinion dynamics on network *A* corresponds to that of the M-model, where the state of each agent can take one of four possible values (*S* = −2,−1, 1, 2), describing its level of agreement on a given issue. The likelihood to become an extremist (*S* = ±2) or a moderate (*S* = ±1) is controlled by a reinforcement parameter *r* ≥ 0. The decision making dynamics on network *B* is akin to that of the Abrams-Strogatz model, where agents can be either in favor (*S* = +1) or against (*S* = −1) the issue. The probability that an agent changes its state is proportional to the fraction of neighbors that hold the opposite state raised to a power *β*. Starting from a polarized case scenario in which all agents of network *A* hold positive orientations while all agents of network *B* have a negative orientation, we explore the conditions under which one of the dynamics prevails over the other, imposing its initial orientation. We find that, for a given value of *β*, the two-network system reaches a consensus in the positive state (initial state of network *A*) when the reinforcement overcomes a crossover value r*(*β*), while a negative consensus happens for *r* < r*(*β*). In the *r* − *β* phase space, the system displays a transition at a critical threshold *β_c_*, from a coexistence of both orientations for *β* < *β_c_* to a dominance of one orientation for *β* > *β_c_*. We develop an analytical mean-field approach that gives an insight into these regimes and shows that both dynamics are equivalent along the crossover line (r*, β*).

## 1 Introduction

The study of complex networks has become a matter of great interest to scientists, due to the large number of real systems that evolve on top of these kind of topological structures, such as human societies, climate, transportation and physiological systems. For many years researchers were focused on studying the topology of isolated networks, and its effect on different dynamics [1–13]. However, it is known that many real-world systems are not isolated but they interact with each other, and they are well described by a multilayer system of interconnected networks [14–17], where nodes belonging to different networks interact. A different multilayer context is that of multiplex networks, in which the same nodes exist –and represent the same entity– in different network layers (see [15] and references therein). The study of multilayer systems allows to understand the interplay between complex networks, and how this affects the processes propagating on them, e.g, synchronization [18, 19], diffusion [20], percolation [21–26] and epidemic spreading [27–35]. Within the context of social science, the study of social phenomena on multilayers is relatively new [15]. Multilayer networks have recently been applied to study opinion dynamics [36], a topic that has many analogies with the dynamics of species competition [37], and that has been extensively studied by statistical physicists. In reference [38], Halu et al. use two interacting networks to describe two political parties that compete for votes in an election. Diakonova et al. explored in [39] the dynamics of the voter model for opinion formation on a bilayer network system with coevolving links, and also studied in [40] the reducibility of the voter model on a two-layer multiplex to a single layer system.

The process of opinion formation may affect and depend on other social processes like decision making [41], due to the relationships between the individuals taking part in each of these two processes. For instance, people in a civil society discuss and form their opinions on a given issue, such as the legalization of the marriage between people of the same sex. However, the decision on whether the same-sex marriage law is approved or not is discussed and finally taken in a legislative body, such as the Congress. As a consequence, these two social groups –society and Congress– influence each other, as congressmen form part and interact with members of the society and, at the same time, people in the society are influenced by what the Congress is deciding.

In this article we investigate the interaction between two social dynamics, one for opinion formation and the other for decision making, that take place on two interconnected networks. The dynamics for opinion formation corresponds to that of the model proposed by La Rocca *et. al* [42], to which we refer as the M-model. This model possesses 2*M* different states describing the spectrum of possible opinion orientations on a given issue, from totally against (state *S* = −*M*) to totally in favor (*S* = *M*), with some moderate opinions between these extreme values. The M-model explains the phenomena of polarization in a population of individuals that evolve under pairwise interactions, by implementing two main social mechanisms for opinion formation, compromise and persuasion [43–45]. The decision making dynamics is akin to that of the Abrams-Strogatz (AS) model [6, 46] (originally introduced to study language competition), where agents can choose between only two possible choices, to be either in favor (*S* = +1) or against (*S* = −1) the issue. Each agent may change its decision by a mechanism of social pressure, in which the probability of switching its present choice increases non-linearly with the number of neighbors that make the opposite choice. In this work, we set the system to explore a hypothetical polarized scenario where, initially, all the agents in the opinion network are in favor of the issue (positive orientations), while all the agents in the decision network are against (negative orientations). By means of this simple model we address the following questions: under which conditions the opinion dynamics is able to influence and reverse the initial orientation of the decision network? Which dynamics is stronger and prevails in the long run? We need to mention that the present proposed model on two interacting networks has some analogies with models of coupled spin systems previously studied to describe the phase diagram of orientational glasses [47, 48]. We also notice that, even though we use in this study the M-model and the AS model for their simplicity, other social models can be implemented as well to explore the interplay between opinion and decision making processes.

The rest of the paper is organized as follows. In Section 2 we introduce the model, describing the topology of interactions as well as the dynamics that runs over each network. Results from numerical simulations of the model are presented in Section 3, where we show that there are three possible final states: a coexistence of both orientations (neither dynamics dominates), a positive consensus (opinion dynamics domination) and a negative consensus (decision dynamics domination). Then, in Section 4 we develop a mean field approach that allows to explain the qualitative behavior of the system, and shows that both dynamics behave equivalently for some particular choice of the parameters. Finally, in Section 5 we summarize and discuss our findings.

## 2 The Model

In our model we consider two interconnected networks, denoted by networks *A* and *B*, each with the same number of nodes *N* and intranetwork degree distribution *P*(*k*), which represents the fraction of nodes connected to *k* other nodes within the same network. We also consider pairwise interconnections, that is, each node is connected to one randomly chosen node in the other network, through an internetwork link. Therefore, a node with *k* intranetwork links and one internetwork link is connected to a total of *k* + 1 neighbors: *k* from the same network and 1 from the other network. In order to keep the internetwork topology as simple as possible, we allow each node to have only one internetwork link. However, the qualitative behavior of the system is expected to be the same if other more complex internetwork patterns are used. In this particular topology, nodes and links represent agents and their social interactions, respectively, and thus the terms “nodes” and “agents” are used alternatively along the article.

**Fig 1:**
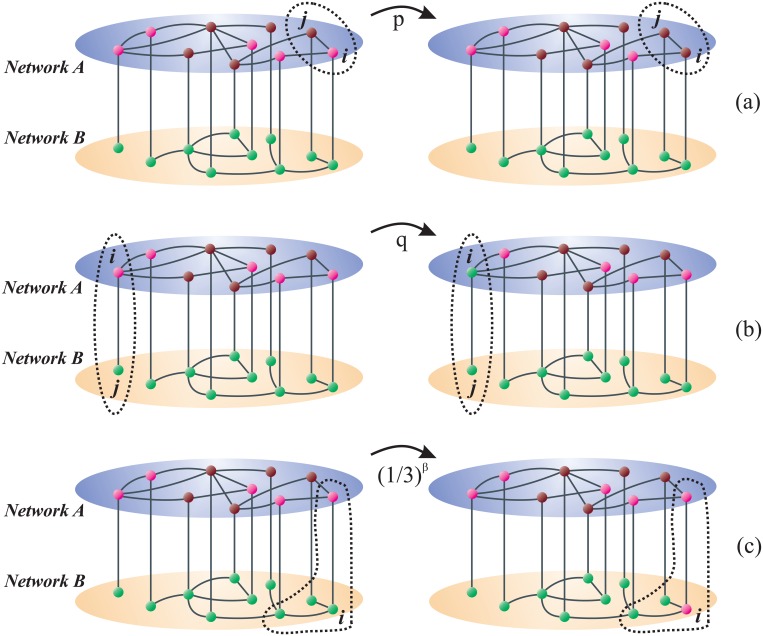
Schematic representation of two interconnected networks with *N* = 10 nodes in each layer. The dynamics on the top network *A* (blue) obeys the M-model, while the dynamics on the bottom network *B* (beige) is akin to that of the Abrams-Strogatz model. The colors of the nodes correspond to different opinion states: *S* = 1 (pink), *S* = 2 (burgundy) and *S* = −1 (green). The figures from the left (right) represent the situation before (after) the chosen node changes its state. (a) A moderate node *i* (*S_i_* = 1) and a extremist neighbor *j* (*S_j_* = 2) in network *A* are chosen. Then *i* becomes extremist with probability *p* (*S_i_* = 1 → 2). (b) A moderate positive node *i* (*S_i_* = 1) in network *A* and a negative neighbor *j* (*S_j_* = −1) in network *B* are chosen. Then *i* becomes a negative moderate with probability *q* (*S_i_* = 1 → −1). (c) The chosen node *i* belongs to network *B* and is a negative moderate (*S_i_* = −1) with total degree *k_i_* = 3 (internal and external degrees *k* = 2 and *k* = 1, respectively). Then it changes orientation (*S_i_* = −1 → 1) with probability (1/3)^*β*^.

The dynamics on network *A* corresponds to that of the M-model [42] with *M* = 2, where only one random agent updates its state at each time step, unlike the original version of the model where two randomly chosen agents can change their states. The opinion state of each agent is represented by an integer number *S^A^* with four possible values *S^A^* = −2, −1, 1 or 2, where the sign of *S^A^* indicates its opinion orientation and its absolute value |*S^A^*| measures the intensity of its opinion. Thus, *S^A^* = 2 and *S^A^* = −2 represent positive and negative extremists, that is, people totally in favor or against the issue, respectively, whereas *S^A^* = 1 and *S^A^* = −1 describe moderate opinions from each side. In a single step of the dynamics, an agent and one of its neighbors are chosen at random. A moderate agent is persuaded by a same-orientation neighbor to become an extremist with persuasion probability *p* (|*S^A^*| = 1 → 2 transition), while an extremist agent becomes moderate (|*S^A^*| = 2 → 1) and a moderate agent changes orientation (*S^A^* = ±1 → ∓1) with compromise probability *q* when they interact with an opposite-orientation neighbor [see Fig 1A and 1B]. As we choose *p* + *q* = 1 and the M-model dynamics depends on the relative ratio *r* ≡ *p*/*q* between the probabilities to become an extremist or a moderate [42], we can express both probabilities *p* = *r*/(1 + *r*) and *q* = 1/(1 + *r*) as function of *r*. The parameter *r* measures the strength of *reinforcement* in the opinion orientation, i e., the tendency of same-orientation neighbors to adopt a more extreme viewpoint as they persuade each other. Thus, for large values of *r* most agents tend to keep their opinions close to the extreme values *S* = 2 or *S* = −2, while for small *r* opinions tend to remain close to the moderate values *S* = 1 or *S* = −1. This model was studied on single fully connected networks in [42], where it was shown that the system reaches a quasistationary state whose features depend on *r*. A polarized state is obtained for *r* > 1 (persuasion larger than compromise), where agents’ opinions are driven to the extreme values *M* and −*M*, and thus the distribution of opinions becomes “U-shaped”, with peaks at *M* and −*M*. A centralized state is observed for *r* < 1 (compromise larger than persuasion), in which most agents hold opinions close to the moderate values 1 and −1. The final state in the long time limit corresponds to an opinion consensus in either state *M* or −*M* (all agents in the same state *M* or −*M*), depending on whether there is an initial majority of positive or negative agents, respectively. When the system reaches this completely ordered state opinions cannot longer evolve, and thus we say that consensus is an absorbing state of the dynamics.

The decision making dynamics of network *B* is similar to that of the AS model [6, 46], where each agent can choose to be either in favor (choice state *S^B^* = +1) or against (choice state *S^B^* = −1) the given issue. This non-linear version of the voter model [5] implements the peer pressure as a social mechanism to change an attitude or behavior: an agent can change its mind and reverse its decision with a probability equal to a power *β* (the *volatility*) of the fraction of its opposite-choice neighbors [see Fig 1C]. The volatility exponent *β* measures how prone a node is to changing state, from very likely for *β* ≃ 0 to very unlikely for *β* ≫ 1. The dynamics of the AS model was extensively studied in single topologies, including fully connected networks as well as complex networks and lattices (see [6] and references therein). This model exhibits a transition from a coexistence of both states (even mix of +1 and −1 agents) to a consensus in either state +1 or −1, as *β* overcomes a threshold value *β_c_* ≃ 1 that is slightly sensitive to the topology of interactions and the symmetry between both states. The coexistence regime of non-consensus is quasistationary in finite systems, because finite-size fluctuations eventually drive the system to one of the two absorbing consensus states.

A distinctive feature of both the M-model and the AS model on single topologies is that their consensus states are attractive. Therefore, starting from a configuration where all agents have the same state *S* = ±*M* in the M-model (or *S* = ±1 in the AS model), we can introduce a small perturbation by changing the states of a few agents at random, and check that the dynamics quickly brings the system back to the initial consensus state. The stability of the consensus state in the M-model increases with *r*, as agents have a larger probability to adopt and keep their initial extreme opinions. For its part, the stability of consensus in the AS model increases with *β*, as agents are less likely to change their choices. Then, an interesting situation happens when these two models are coupled and start from opposite oriented consensus states, given that each dynamics tries to bring the entire two-network system to its own initial state. The interplay between the two dynamics would eventually drive the system to one of the two initial consensus states, and thus we can interpret this outcome as the prevalence of one dynamics over the other. We expect that the final result depends on the relative values of parameters *r* and *β*, which are proportional to the “strength” of the M-model and the AS model, respectively.

Since we are interested in studying which dynamics dominates in the long run we initially set all nodes in network *B* to state *S^B^* = −1, while in network *A* we randomly assigned state *S^A^* = 2 to *N*/2 nodes and state *S^A^* = 1 to the other *N*/2 nodes (all nodes positively oriented but with different intensities). Then, at each time step of length Δ*t* = 1/2*N*, a node *i* is chosen at random from the two networks and its state *S_i_* is updated according to whether *i* belongs to network *A* or *B*:

1. *Node i in network A*: one of its *k_i_* + 1 neighbors, node *j* with state *S_j_*, is randomly chosen. If *i* and *j* share the same orientation (*S_i_* *S_j_* > 0), then with probability *p* node *i* adopts an extremist state if it is a moderate (*S_i_* = ±1 → ±2), and, independently of the interaction, remains extremist if it is already an extremist (*S_i_* = ±2 → ±2) [see Fig 1A]. If *i* and *j* have opposite orientations (*S_i_* *S_j_* < 0), with probability *q* node *i* becomes moderate if it is an extremist (*S_i_* = ±2 → ±1), or changes orientation if it is a moderate (*S_i_* = ±1 → ∓1) [see Fig 1B].
2. *Node i in network B*: the state of *i* changes with probability
  $$P_{B}\left(S_{i}\mapsto -S_{i}\right)={\left(\frac{n}{k_{i}+1}\right)}^{\beta },$$ (1)
  where *n* is the number of neighbors of *i* with opposite orientation than *i*, and *β* ≥ 0 is the volatility.

In the next Section we explore the behavior of the model using *β* and *r* as external control parameters.

## 3 Simulation Results

We studied the model described in Section 2 by means of Monte Carlo simulations using two interconnected degree-regular random networks (DR) of degree *μ* = 5 and *N* nodes each. We implemented the Molloy-Reed algorithm [49] to build the networks, where each node is connected to *μ* random nodes in the same network, and to one random node in the other network. Starting from a polarized situation that consists of setting all nodes in network *A* to positive states and all nodes in network *B* to negative states, we let the system evolve following the M-model and the AS dynamics described in Section 2 for networks *A* and *B*, respectively. We investigated how the steady state of the system depends on the opinion reinforcement *r* and volatility *β* that control, respectively, the strength of agents’ persuasion in network *A* and the likelihood that an agent in network *B* changes its decision. Because we were particularly interested in studying whether the dynamics in network *A* prevails over the dynamics in network *B* (or vice versa), we run many independent realizations of the dynamics and calculated the probability *P*_+_ that the entire two-network system reaches a + consensus, that is, the initial orientation adopted by network *A*. We consider that the system reaches consensus when all nodes of both networks have the same orientation (either positive + or negative −). Notice that, for instance, states *S* = 2 and *S* = 1 are both considered as positively oriented. The probability *P*_+_ was estimated as the fraction of realizations that ended in a + consensus. Given that each separate model always reaches consensus in a finite network –as explained in Section 2–, one can check that the probability of a − consensus in the entire system is *P*_−_ = 1 − *P*_+_.

**Fig 2:**
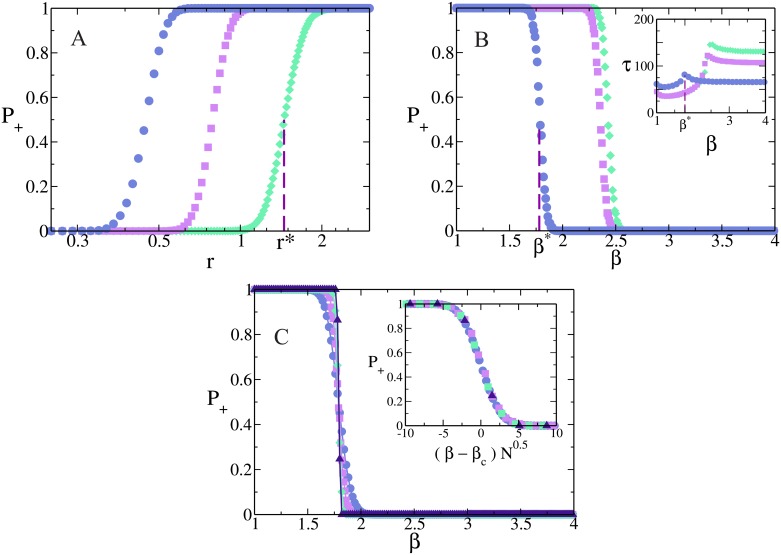
Probability of positive consensus *P*_+_ in a system of two interconnected networks *A* and *B*. Initially, all nodes in network *A* (*B*) are positive (negative). (a) *P*_+_ as function of *r* = *p*/*q* on a log-linear scale, for networks of size *N* = 2048 nodes and *β* = 2.0 (∘), 2.25 (□) and 2.5 (◇). At the crossover point r*(*β*) is *P*_+_ = 1/2 (vertical dashed line shown for *β* = 2.5 only). (b) *P*_+_ vs *β* for *r* = 0.25 (∘), 1.0 (□) and 1.2 (◇). At β*(*r*) is *P*_+_ = 1/2 (vertical dashed line for *r* = 0.25). Inset: mean consensus time *τ* vs *β*, for the same parameter values, showing a maximum at β*. (c) *P*_+_ vs *β* for *r* = 0.25 and network sizes *N* = 512 (∘), 2048 (□), 8192 (◇) and 32768 (△). Inset: the curves collapse when the *x*-axis is rescaled by $\left(\beta -\beta^{*}\right)\sqrt{N}$. All numerical results correspond to an average over 10^4^ independent realizations on degree-regular random networks of degree *μ* = 5.

In Fig 2A we plot *P*_+_ as a function of *r* for three different volatilities *β*. We observe that *P*_+_ increases abruptly from 0 to 1.0 when *r* overcomes a crossover value r*(*β*), determined as the symmetric point where *P*_+_ = 1/2. This means that for large reinforcement *r* > r* network *A* imposes its initial orientation to network *B*, and thus the dynamics of the M-model prevails over the AS dynamics. The opposite happens for low reinforcement *r* < r*, where the initial orientation of network B prevails, and thus the dynamics of the AS model is stronger than that of the M-model. An interpretation of these results can be given in terms of the response of the M-model to a variation in *r*. As described in Section 2, the initial positive consensus in the M-model on network *A* becomes more stable as *r* increases. Then, it turns out that for very small values of *r* the initial A-consensus is very unstable, and all nodes in network *A* quickly adopt the negative states hold by nodes in network B, driving the entire system to a − consensus in most realizations (*P*_+_ ≃ 0). In the opposite limit of very large values of *r*, the initial A-consensus is very stable, thus most A-nodes keep their initial positive states while B-nodes change their states to positive, and the entire system reaches a + consensus in most realizations (*P*_+_ ≃ 1). Finally, for intermediate values of *r* some realizations end in a + consensus while the rest end in a − consensus, leading to the sigmoidal shape of *P*_+_ vs *r* in Fig 2A.

In Fig 2B we plot *P*_+_ vs *β* for three values of *r*. We can see a crossover from + to − consensus at a value β*(*r*), where *P*_+_ = 1/2, in a similar fashion to the crossover with *r* described above. For *β* > β* network *B* imposes its initial orientation to network *A*, while for *β* < β* the opposite happens. This behavior can be explained using arguments similar to those used above to explain the crossover of *P*_+_ at r*. As *β* increases from small values, the initial − consensus state of network *B* gains stability, continuously increasing the probability that the system reaches a − consensus or, equivalently, decreasing *P*_+_. The reason why curves start at *β* = 1 is because for low values of *β* consensus states are never observed in the simulations, even though finite systems must reach consensus as we noted before. As we shall see when we analyze other observable like the magnetization, for *β* < *β_c_* ≃ 0.86 the system falls in an active steady state with + and − orientations coexisting in both networks but, after a long time, consensus is eventually achieved by fluctuations. Consensus times in this regime are extremely long for the system sizes we used, and thus consensus is never achieved in a reasonable computer time. Indeed, we have run simulations on small enough networks and checked that an absorbing state is always reached. As we shall explain, this quasistationary non-consensus state is related to the coexistence dynamics observed in the AS model for *β* < *β_c_* ≃ 1.

Fig 2C shows *P*_+_ vs *β* for *r* = 0.25 and different network sizes *N*. We can see that the crossover becomes sharper as *N* increases, with a slope at β* that diverges as $\sqrt{N}$, as the data collapse in the inset of Fig 2C shows. In the inset of Fig 2B we show the mean time *τ* to reach the consensus state as a function of *β*, for the values of *r* of the main Fig 2B. We observe that *τ* has a peak at β*, which is consistent with the fact that at the crossover point the system can reach either + or − consensus with the same probability 1/2, suggesting that large fluctuations lead the system to the final state. In Section 4 we give an insight into this last behavior and show that the breaking in the symmetry of the system at β* eventually happens after a long time, when finite-size fluctuations make the system overcome a potential barrier. Below β* the M-model in network *A* seems to control the dynamics of the system –as there is a + consensus in both networks–, and thus *τ* is determined by the time it takes for network *B* to reach a + orientation from an initial − orientation, which increases with *β*. But above β* the opposite happens: network *B* rules the dynamics, and thus *τ* is related to the time that network *A* takes to go from a positive to a negative orientation. This observation is in agreement with the fact that *τ* approaches a constant value as *β* becomes large, given that the M-model is independent of *β*, and then so is *τ*.

In order to explore the behavior of the system for a wider range of *β*, we study the magnetization in networks *A* and *B*, *m*_*A*_ and *m*_*B*_, respectively, at the steady state. The magnetization in network *ℓ* (*ℓ* = *A*, *B*) at time *t* is defined as

$$m_{\ell }=\sigma_{\ell }^{+}-\sigma_{\ell }^{-},$$ (2)

with *m*_ℓ_ ≡ *m*_ℓ_(*t*), $\sigma_{\ell }^{+}\equiv \sigma_{\ell }^{+}\left(t\right)$, $\sigma_{\ell }^{-}\equiv \sigma_{\ell }^{-}\left(t\right)$, and where $\sigma_{\ell }^{+}$ and $\sigma_{\ell }^{-}$ are the fractions of nodes with + and − state, respectively, in network *ℓ* at time *t*.

**Fig 3:**
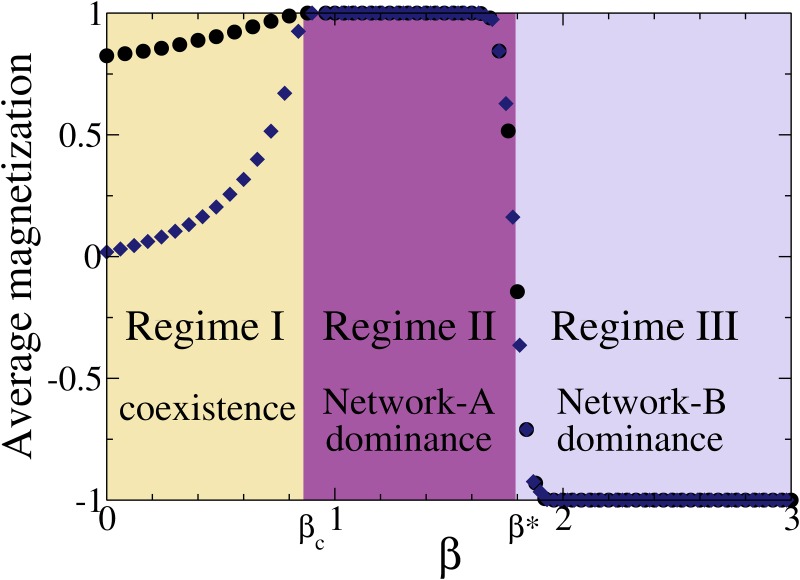
Average magnetization at the steady state 〈*m*_*A*_〉 (circles) and 〈*m*_*B*_〉 (diamonds) in networks*A* and *B*, respectively, as a function of *β*, for*r* = 0.25. Below the critical threshold *β_c_* ≃ 0.86 the system remains in a disordered state where both + and − orientations coexist (Regime I), while above *β_c_* the system reaches an ordered state of consensus (Regimes II and III). The point β* denotes the crossover between Regimes II and III, characterized by a positive and negative consensus, respectively. Numerical results correspond to two DR random networks of degree *μ* = 5 and size *N* = 2048 each, averaged over 10^4^ independent realizations.

As we mentioned above, consensus in one of the two orientations is only observed in the simulations when *β* is above a critical value *β_c_* ≃ 0.86, while for *β* < *β_c_* the system remains in an active steady state with both positive and negative orientations coexisting. This means that, in the 0 ≤ *β* < *β_c_* region, magnetizations *m*_*A*_ and *m*_*B*_ in a single realization fluctuate around two different stationary values that are neither 1.0 nor −1.0. This is shown in Fig 3, where we plot the average magnetization over many realizations at the steady state in each network, 〈*m*_*A*_〉 and 〈*m*_*B*_〉, as a function of *β* ≥ 0, for *r* = 0.25 and *N* = 2048. We can distinguish three different regimes. In the first regime (denoted by regime I), we see that 〈*m*_*A*_〉 (〈*m*_*B*_〉) increases from 0.8 (0.0) to 1.0 (1.0) in the range from *β* = 0 to *β_c_* ≃ 0.86. That is, there is a majority of nodes with positive orientation in network *A*, while in network *B* the coexistence is more even. We note that, strictly speaking, this coexistence regime is stable only in the thermodynamic limit, where the system remains forever in a stationary state of non-consensus. As stated before, in finite systems the steady state lasts for very long times, but fluctuations ultimately drive the system to an absorbing consensus state.

Above *β_c_* the system reaches a positive consensus 〈*m*_*A*_〉 = 〈*m*_*B*_〉 = 1.0 (network-*A* dominance) for *β_c_* < *β* < β* (denoted by regime II), and a negative consensus 〈*m*_*A*_〉 = 〈*m*_*B*_〉 = −1.0 (network-*B* dominance) for *β* > β* (denoted by regime III). In regimes II and III close to β*, an average value of the magnetization different from 1 and −1 means that some fraction of the realizations ended in a positive consensus and the rest in a negative consensus.

The values of *β_c_* and β* are very different in nature. While *β_c_* denotes a critical point from a disordered phase (regime I) to an ordered phase (regimes II and III), β* denotes a crossover point within the ordered phase, which separates the two dominance regions. We also note that the order-disorder transition at *β_c_* is related to the same type of transition observed in the AS model, explained in Section 2. It seems that the coexistence phase in the isolated AS dynamics is very robust, and the coupling to the M-model produces only a shift in the critical value, from *β_c_* ≃ 1 to *β_c_* ≃ 0.86.

**Fig 4:**
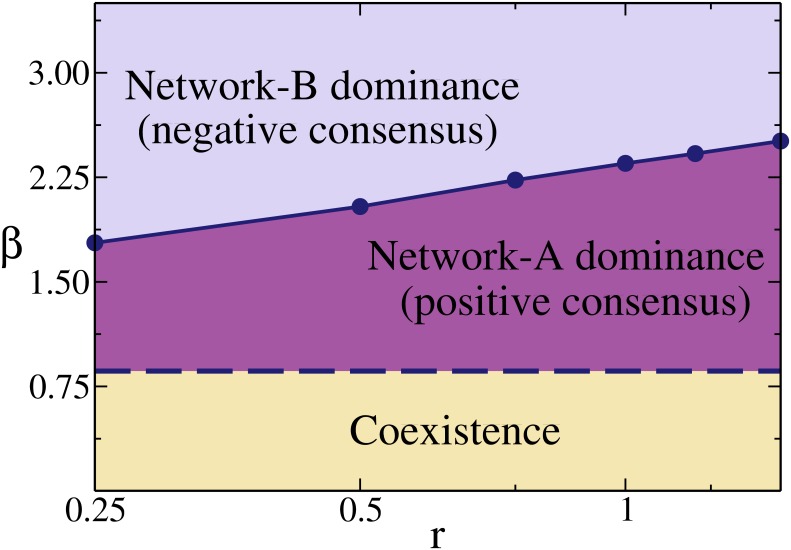
Reinforcement-volatility (*r* − *β*) phase diagram on a log-linear scale for a two-network system with the same parameters as in Fig 2. Solid circles correspond to the crossover points (r*, β*) between network-A and network-B dominance regions, while the dashed line represents the transition point *β_c_* ≃ 0.86 between coexistence and consensus.

Fig 4 shows the phase diagram of the system in the *r* − *β* plane, on a log-linear scale. We observe that the crossover point β* increases very slowly (logarithmically) with r*. Therefore, starting from a point (*r*, *β*) inside the B-dominance region, an exponentially large increase in *r* must be done to take the system to the A-dominance region. In other words, for a small change in the volatility of the decision making dynamics of network B, the dynamics of network *A* has to increase its opinion reinforcement by a large amount, in order to impose its initial orientation.

In the next Section we develop a theoretical approach that allows to explain the qualitative behavior of the system in the three regimes. Even though this approach assumes that the system is infinitely large, is able to capture most of the phenomenology observed in the simulations, which are for finite networks.

## 4 Mean Field Approach

As we showed in Section 3, the system exhibits three different regions in the *r* − *β* phase space: a coexistence of + and − nodes for *β* below a critical value *β_c_*, a + consensus for *β_c_* < *β* < β*(*r*) where the M-model in network *A* dominates, and a − consensus for *β* > β*(*r*) where the AS model in network *B* dominates. In order to understand the role of *β* and *r* in the behavior of the system in these three regions, we study in this Section the evolution of the system within a mean-field approach. To be specific, we write and analyze approximate equations for the time evolution of the magnetization in each network.

As the system is symmetric at β*, where consensus is equally reached in both opinion orientations, we assume that the dynamics of both models are equivalent at β* and, therefore, we consider the M-model as an AS model with a volatility exponent β*. Roughly speaking, we can think of mapping the four-state M-model into a two-state AS model by combining *S* = 1 and *S* = 2 states into a single + state and *S* = −1 and *S* = −2 into a single − state, and considering effective transition probabilities between + and − states that are non-linear functions of the fractions *σ*^+^ and *σ*^−^ of + and − neighbors of a given node, respectively. For instance, the effective transition probability of a node *i* from − to + can be written as (*σ*^+^)^β*^, where *σ*^+^ is the fraction of *i*’s neighbors in the opposite state + (*S* = 1 and *S* = 2 states). Even though it is difficult to obtain the exact value of the exponent β*, one can show that β* should be larger than 1.0 using the following heuristic argument. The effective transition probability from − to + states involves single jumps from nodes in state *S* = −1 to state *S* = 1, whose probability is proportional to the fraction of + neighbors *σ*^+^, and also double jumps from nodes in state *S* = −2 to *S* = −1 and then to *S* = 1, with a probability proportional to (*σ*^+^)^2^. Combining these two types of transitions in the entire network results in an effective probability with an exponent 1.0 < β* < 2.0.

The advantage of mapping the four-state M-model into a two-state model is that it allows to reduce the original two-network system –where the M-model interacts with the AS model– to a simpler system consisting on two interacting AS models, which can be studied analytically. Even though these two systems are not exactly the same because the mapping of the M-model into the AS model is only approximate, we shall see that both systems share the same phenomenology, with results that are in qualitative agreement with the simulation results of Section 3, including a transition and a crossover between the different regimes.

Based on these assumptions, we study a system that consists of two interconnected networks *A* and B, where an AS dynamics with fixed volatility *α* = β* runs on network *A* (representing the M-model), and another AS dynamics with variable volatility *β* runs on network *B*. We start by deriving an approximate equation for the time evolution of the magnetization $m_{\ell }=\sigma_{\ell }^{+}-\sigma_{\ell }^{-}$ in network *ℓ* (*ℓ* = *A*, *B*), where $\sigma_{\ell }^{S}$ is the fraction of nodes with state *S* (*S* = +, −) in each network, which obeys the normalization condition $\sigma_{\ell }^{+}+\sigma_{\ell }^{-}=1$. At each time step Δ*t* = 1/2*N*, a node *i* in network *A* with state *S* is chosen with probability $\sigma_{A}^{S}/2$, and switches to state −*S* with probability *P*_*A*_(*S* → −*S*), changing *m*_*A*_ by Δ*m*_*A*_ = −2*S*/*N*. Then, the average change in the magnetization of network *A* can be written as

$$\frac{dm_{A}}{dt}=\frac{1}{1/2N}\left[\frac{\sigma_{A}^{-}}{2}\;P_{A}\left(-\to +\right)\frac{2}{N}-\frac{\sigma_{A}^{+}}{2}\;P_{A}\left(+\to -\right)\frac{2}{N}\right].$$ (3)

Using Eq (1) for the switching probability, *P*_*A*_ can be approximated as

$$P_{A}\left(S\to -S\right)≃{\left(\frac{\langle n_{A}\rangle }{\mu +1}\right)}^{\alpha },$$ (4)

where 〈*n*_*A*_〉 is the expected number of neighbors of node *i* with opposite state −*S*, and *μ* + 1 is the total number of neighbors. Within a mean-field approach that neglects nearest-neighbor correlations (node approximation), a neighbor of *i* in network *A* (*B*) is in state −*S* with probability $\sigma_{A}^{-S}$ ($\sigma_{B}^{-S}$) and, therefore, the expected number of neighbors with state −*S* of *i* can be estimated as

$$\langle n_{A}\rangle ≃\mu \;\sigma_{A}^{-S}+\sigma_{B}^{-S}.$$ (5)

Using Eqs (4) and (5) and expressing the densities of states in terms of the magnetization $\sigma_{A}^{S}=\left(1+S\;m_{A}\right)/2$, Eq (3) can be written as

$$\frac{dm_{A}}{dt}=\frac{\left(1-m_{A}\right)}{2^{\alpha }{\left(\mu +1\right)}^{\alpha }}{\left[\mu \left(1+m_{A}\right)+1+m_{B}\right]}^{\alpha }-\frac{\left(1+m_{A}\right)}{2^{\alpha }{\left(\mu +1\right)}^{\alpha }}{\left[\mu \left(1-m_{A}\right)+1-m_{B}\right]}^{\alpha },$$ (6)

and a corresponding equation can be derived for *m*_*B*_,

$$\frac{dm_{B}}{dt}=\frac{\left(1-m_{B}\right)}{2^{\beta }{\left(\mu +1\right)}^{\beta }}{\left[\mu \left(1+m_{B}\right)+1+m_{A}\right]}^{\beta }-\frac{\left(1+m_{B}\right)}{2^{\beta }{\left(\mu +1\right)}^{\beta }}{\left[\mu \left(1-m_{B}\right)+1-m_{A}\right]}^{\beta }.$$ (7)

Eqs (6) and (7) can be rewritten in the form of a time-dependent Ginzburg-Landau equation [6]

$$\begin{array}{ccc} \frac{dm_{A}}{dt} & = & -\frac{\partial V_{A}}{\partial m_{A}}\;, \end{array}$$ (8)

$$\begin{array}{ccc} \frac{dm_{B}}{dt} & = & -\frac{\partial V_{B}}{\partial m_{B}}\;, \end{array}$$ (9)

with potentials *V*_*A*_ ≡ *V*_*A*_(*m*_*A*_, *m*_*B*_) and *V*_*B*_ ≡ *V*_*B*_(*m*_*A*_, *m*_*B*_) given by

$$\begin{array}{ccc} V_{A}= & - & \frac{{\left[(\mu \left(1+m_{A}\right)+1+m_{B}\right]}^{\alpha +1}\left\{\mu \left[2+\left(\alpha +1\right)\left(1-m_{A}\right)\right]+1+m_{B}]\right\}}{2^{\alpha }{\left(\mu +1\right)}^{\alpha }\mu^{2}\;\left(\alpha +1\right)\;\left(\alpha +2\right)}\\ & - & \frac{{\left[(\mu \left(1-m_{A}\right)+1-m_{B}\right]}^{\alpha +1}\left\{\mu \left[2+\left(\alpha +1\right)\left(1+m_{A}\right)\right]+1-m_{B}]\right\}}{2^{\alpha }{\left(\mu +1\right)}^{\alpha }\mu^{2}\;\left(\alpha +1\right)\;\left(\alpha +2\right)},\;\;\;\;\;\; \end{array}$$ (10)

$$\begin{array}{ccc} V_{B}= & - & \frac{{\left[(\mu \left(1+m_{B}\right)+1+m_{A}\right]}^{\beta +1}\left\{\mu \left[2+\left(\beta +1\right)\left(1-m_{B}\right)\right]+1+m_{A}]\right\}}{2^{\beta }{\left(\mu +1\right)}^{\beta }\mu^{2}\;\left(\beta +1\right)\;\left(\beta +2\right)}\\ & - & \frac{{\left[(\mu \left(1-m_{B}\right)+1-m_{A}\right]}^{\beta +1}\left\{\mu \left[2+\left(\beta +1\right)\left(1+m_{B}\right)\right]+1-m_{A}]\right\}}{2^{\beta }{\left(\mu +1\right)}^{\beta }\mu^{2}\;\left(\beta +1\right)\;\left(\beta +2\right)}.\;\;\;\;\;\; \end{array}$$ (11)

This formalism is very useful for visualizing the system’s evolution, as each magnetization evolves towards the minimum of its associated potential. However, unlike it happens in the AS model on a single isolated network [6] where the potential depends on a unique magnetization and is static, the present case has two coupled potentials that vary in time. Indeed, Eq (10) for the potential *V*_*A*_ that rules the evolution of *m*_*A*_ can be interpreted as an explicit function of *m*_*A*_, whose shape is controlled by a time-dependent external parameter *m*_*B*_. Therefore, the shape of *V*_*A*_ varies with time through *m*_*B*_. An analogous interpretation can be done for *V*_*B*_, which depends on *m*_*A*_. Thus, within this approximate mathematical formalism represented by the coupled system of Eqs (8) and (9), the interplay between both networks enters through the potentials *V*_*A*_ and *V*_*B*_, which interact and co-evolve in time.

We now explore the behavior of the two networks by studying the evolution of the magnetization described by Eqs (6) and (7), and using the potential formalism. For network *A* we set the volatility value *α* = β* = 1.78 corresponding to the crossover point for *r* = 0.25 calculated in Section 3, and vary the volatility *β* in network *B*. The phenomenology described below is qualitatively the same for the values of *α* that correspond to the other values of *r* used in Fig 4.

**Fig 5:**
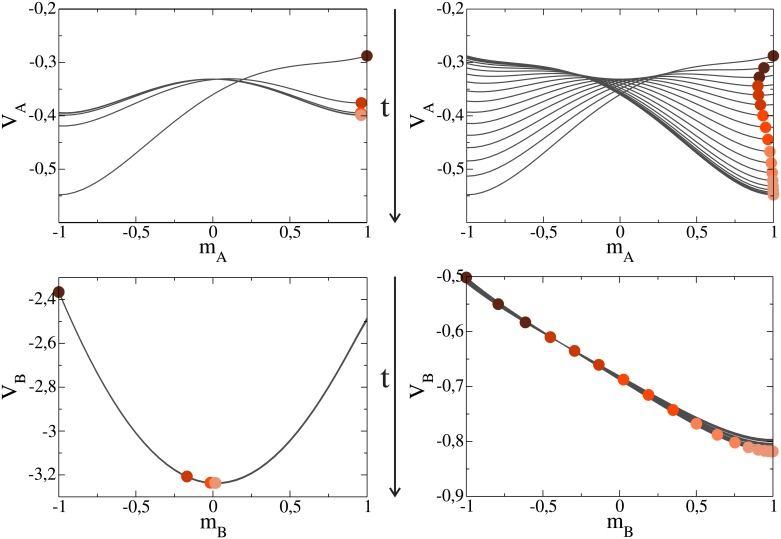
Potentials *V*_*A*_ and *V*_*B*_ (solid lines) as a function of the magnetizations *m*_*A*_ and *m*_*B*_ in networks *A* and B, respectively, at different times, obtained from Eqs (10) and (11). The degree in both networks is *μ* = 5. The volatility in network *A* is *α* = 1.78, while in network *B* is *β* = 0.1 (left panel) and *β* = 1.2 (right panel). Circles correspond to the values of the magnetizations at different times, starting from the dark topmost circle at *t* = 0 and ending at the lightest circle for long times. Vertical arrows indicate the time direction. Plots in the left panel show the coexistence regime I, while plots in the right panel describe the positive consensus regime II.

**Fig 6:**
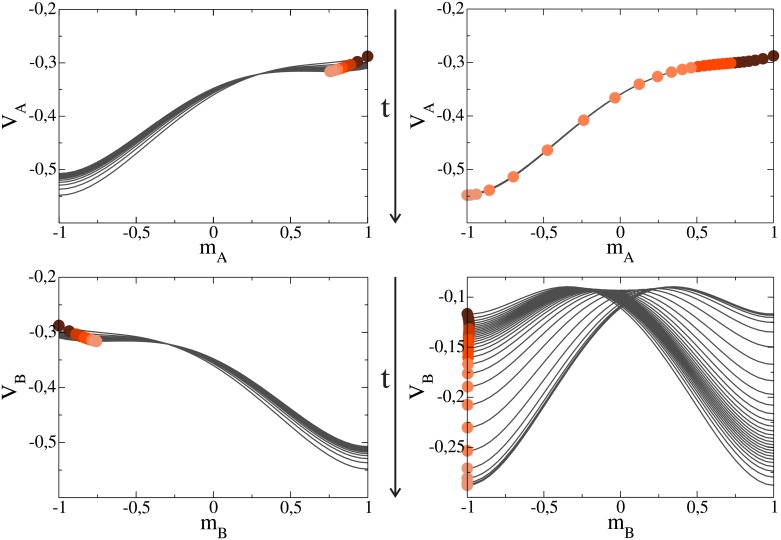
Potentials *V*_*A*_ and *V*_*B*_ as in Fig 5, but for volatility values *β* = *α* = 1.78 (left panel) and *β* = 3 (right panel). Plots in the left panel correspond to the crossover point, a symmetric case where the system remains disordered, while plots in the right panel show the negative consensus regime III.

To visualize the trajectories of the magnetization, we plot in Figs 5 and 6 the values of *m*_*A*_ and *m*_*B*_ (circles) and their associated potentials (solid lines) at different times, for various parameter values. Each circle corresponds to the magnetization *m*_*ℓ*_ at a given time *t*, which lies over the potential *V*_*ℓ*_ at the same time *t*, with *ℓ* = *A*, *B*. The intensity of a circle’s color decreases as time increases, starting from *t* = 0 (dark circle) and ending at the lightest color. Drawing the complete shape of the potential helps to understand the trajectory followed by *m*_*ℓ*_, which moves in the direction of the minimum of *V*_*ℓ*_. The values of *m*_*A*_ and *m*_*B*_ were obtained by integrating numerically Eqs (6) and (7), while the potential *V*_*A*_ at a given time *t* was drawn by replacing the value of *m*_*B*_ into Eq (10), and similarly for *V*_*B*_.

Fig 5 (left) shows the behavior in the coexistence regime I, for *β* = 0.1 < *β_c_* ≃ 0.86. As we can see, the magnetization in network *B* evolves from *m*_*B*_ = −1.0 at *t* = 0 to the minimum at *m*_*B*_ ≃ 0 for long times (approximately 51% of positive agents), while *m*_*A*_ in network *A* starts at 1.0 and reaches the stationary value *m*_*A*_ ≃ 0.95 close a positive consensus. This result is in agreement with the one found from simulations for small *β* ≤ *β_c_* (see Fig 3 for small *β*), where the system remains in a disordered phase with a coexistence of both orientations. The behavior in the positive consensus regime II is quite different [Fig 5 (right)]. There we use *β* = 1.2 that lies between *β_c_* ≃ 0.86 and β* = 1.78. We observe that, as it happens in simulations, both networks reach a positive consensus after a few time steps. While *m*_*A*_ quickly gets trapped in a local minimum that ultimately reaches the value *m*_*A*_ = 1, *m*_*B*_ follows a direct trajectory from *m*_*B*_ = −1 towards a unique minimum at *m*_*B*_ = 1. The critical value of *β* that separates regime I (coexistence) from regime II (consensus) was found to be close to 1.0 (not shown), which is quite different from the critical threshold *β_c_* ≃ 0.86 obtained from Monte Carlo simulations. This discrepancy may be due to the fact that the theoretical approach considers an AS model in network *A* (instead of the M-model) and also that Eqs (6) and (7) describe the evolution of *m*_*A*_ and *m*_*B*_ in infinite large systems, as they do not have any terms that take into account finite-size fluctuations.

Fig 6 (left) corresponds to the crossover point *β* = β* = *α*. We see that the magnetizations reach the stationary values *m*_*A*_ ≃ 0.75 and *m*_*B*_ ≃ −0.75, corresponding to a totally symmetric case in which there is an unbalanced coexistence of orientations in each network. Even though the total magnetization *m*_*A*_ + *m*_*B*_ = 0 at the crossover point agrees with the average magnetization obtained from simulations (see Fig 3), there is a discrepancy with simulations results, where consensus in one of the two orientations is always obtained for each individual realization due to finite-size fluctuations. This is because Eqs (6) and (7) describe an infinite large system where fluctuations are neglected and, therefore, the system can never escape from the minimum. Due to the symmetry in both potentials, one would expect a 50% chance to escape towards either consensus state if fluctuations were present, which is consistent with the equal consensus probability in each state *P*_+_ = *P*_−_ = 1/2 shown in section 3. Finally, Fig 6 (right) corresponds to regime III, with *β* = 3 > β*. The behavior in this case is analogous to the one of Fig 5 (right), but with an ultimate negative consensus in both networks (*m*_*A*_ = *m*_*B*_ = −1), in agreement with simulation results of Section 3.

In summary, the theoretical approach of this Section allows to understand the underlying behavior of the system in the different regimes, and gives an insight into why a dynamics prevails over the other.

## 5 Discussion

In this work, we explored the interplay between two different dynamical processes that take place on two interconnected networks *A* and *B*. The dynamics on network *A* corresponds to the one of the M-model for opinion formation with four states (*M* = 2), which implements the mechanisms of compromise and persuasion related by a reinforcement parameter *r*. In network *B* the dynamics is akin to that of the Abrams-Strogatz model for decision making, with two states and a volatility parameter *β*. Both models have positive and negative opinion orientations. We initially set the system in a symmetric condition, where all nodes in network *A* have positive states and all nodes in network *B* have negative states, and studied the conditions under which one of the two dynamics dominates. We found that for a reinforcement larger than a crossover value r*(*β*) the dynamics on network *A* dominates, as a positive consensus is reached in both networks, while the opposite outcome is obtained for *r* < r*(*β*) (network *B* dominates). As we have shown, this is due to the fact that increasing the level of opinion reinforcement in network *A* beyond a value r* produces a large number of positive extremists that are able to resist the change of orientation, imposing their positive orientation to the entire system. Besides, the study of the full *r* − *β* phase space revealed a transition at a critical threshold *β_c_*, from a disordered phase where both orientations coexist to an ordered phase characterized by a consensus of one of the two orientations. We also showed that both dynamics are equivalent along the crossover line (r*, β*) that separates the A-dominance and B-dominance regions, as the consensus probability in either state is the same on the (r*, β*) line. Taking advantage of this symmetry, we developed a mean-field approach for the evolution of the magnetization in each network, using a time-dependent Ginzburg-Landau equation. This approach was able to reproduce qualitatively the different regimes observed in the simulations, and gave an insight into when and how the dominance of one dynamics takes place.

In practical terms, the equivalence between both dynamics means that a rather complex M-model with four opinion states and a reinforcement r* can be mapped to a simpler two-state model with effective transition probabilities given by the exponent β*(r*). This mapping might be very useful to gain an analytical insight into the behavior of the M-model, given that the dynamics of the two-state equivalent model can be understood in terms of its associated Ginzburg-Landau potential. Despite the fact that this result is particular of the opinion and decision making models used in this work, we expect that analogous behaviors can be obtained using other types of dynamics, beyond socially inspired models. As a general remark, one can argue that it is possible to gain a better understanding of a complex and poorly known dynamics by coupling this dynamics to a much simpler a better known two-state model, using two similar interconnected networks as the underlying topology.

While our results are obtained using degree-homogeneous networks, it might be worthwhile to study the system using different network topologies, as real social networks are known to be quite heterogeneous. Even though we limited our internetwork topology to a single random interlink per node, the addition of targeted interlinks connecting specific nodes in both networks may bring new phenomenology. It could also be interesting to investigate how the number of different opinion states in the M-model affects the results, given that a more robust polarized state is expected as the maximum opinion value *M* increases.
